# Water on the Brain

**Published:** 1884-03

**Authors:** 


### WATER ON THE BRAIN

is threatened. Relief must be promptly had or the child is doomed. Put cold compresses or ice-pads on the head, and keep them there ; compel the feet to keep warm, give a warm sitz-bath, and keep at these until the symptoms have abated, and the child sleeps quietly, or is disposed to eat or play.

2. If the lips are apart, with a kind of gritting, there is pain in the belly, and most certainly it has been fed too much or too often.

3. If the nostrils are drawn upward and there is quick breathing, there is pain in the chest ; something is the matter with the lungs.

4. If there is a squinting in the eye, or bluish tint about the lips, and a kind of rotating movement of the eyeballs, convulsions will soon follow ; there is indigestion, and a warm water emetic must be resorted to.

5. If the eyes are unusually dull, or there is an unnatural quickness, with a pearly look of the whites, brain-disease is approaching; give an enema and a dose of castor oil, and feed with regularity. [*Instead of an enema, use "Nelaton's Suppositories for Children."*

